# Impact of esophageal mucosal permeability markers on provocation‐induced esophageal reflexes in high‐risk infants

**DOI:** 10.14814/phy2.15366

**Published:** 2022-06-27

**Authors:** Sudarshan R. Jadcherla, Roseanna Helmick, Kathryn A. Hasenstab, Minna Njeh, Enas Alshaikh

**Affiliations:** ^1^ Innovative Infant Feeding Disorders Research Program Nationwide Children's Hospital Columbus Ohio USA; ^2^ Center for Perinatal Research The Research Institute at Nationwide Children's Hospital Columbus Ohio USA; ^3^ Division of Neonatology Nationwide Children's Hospital Columbus Ohio USA; ^4^ Division of Pediatric Gastroenterology, Hepatology, and Nutrition, Department of Pediatrics The Ohio State University College of Medicine Columbus Ohio USA

**Keywords:** distal baseline impedance, esophageal motility, gastroesophageal reflux disease, pH‐impedance, prematurity

## Abstract

Esophageal distal baseline impedance (DBI) is an indicator of mucosal integrity; lower values suggest increased permeability. Aims were to characterize the (1) effect of DBI category (<900 Ω, 900–2000 Ω, and >2000 Ω) on sensory‐motor characteristics of mid‐esophageal provocation‐induced motility reflexes, and (2) clinical outcomes among high‐risk human infants evaluated for gastroesophageal reflux disease. Symptomatic infants (*N* = 49, 41 ± 3 weeks postmenstrual age) underwent pH‐impedance testing to characterize acid reflux index (ARI) and DBI, and pharyngo‐esophageal manometry to examine upper esophageal sphincter (UES), peristaltic, and lower esophageal sphincter (LES) functions. Sensory‐motor response characteristics included response threshold (ml), occurrence (%), latency (s), duration (s), and magnitude (mmHg) upon mid‐esophageal stimulations (0.1–2.0 ml of air, water, and apple juice). Motility and clinical outcomes were compared among DBI groups. In infants with DBI <900 Ω and 900–2000 Ω (vs. >2000 Ω): (a) Long‐term feeding milestones did not differ (*p* > 0.05); (b) complete peristaltic propagation decreased in 900–2000 Ω (*p* < 0.05), polymorphic waveforms increased in <900 Ω and 900–2000 Ω (*p* < 0.05); (c) media effects were noted with liquids (vs. air) wherein UES and esophageal contractility were prolonged in <900 Ω and 900–2000 Ω (*p* < 0.05), and esophageal sensitivity heightened for <900 Ω with water and for 900–2000 Ω with air (both *p* < 0.05). ARI was not correlated with DBI in infants with chronic lung disease (*r* = 0.05, *p* = 0.82). We conclude that pharyngo‐esophageal motility sensory‐motor characteristics in infants are modified by DBI category. These preliminary findings pave‐the‐way for further physiological testing in convalescing high‐risk infants to ascertain potential mechanisms of airway‐digestive reflex interactions and symptom generation, which may lead to targeted therapies.

## 1 INTRODUCTION

Physicians continue to rely on nonspecific symptoms reported by parents and crib‐side providers to diagnose and treat GERD despite evidence that empirical treatment based on symptoms has a low yield of symptom improvement, albeit with the consequences of pharmacological and non‐pharmacological therapies (El‐Mahdy et al., 2017; Jadcherla et al., 2013; Loots et al., 2014; Slaughter et al., 2016). Diagnosis of GERD in the neonatal intensive care unit (NICU) is extremely variable ranging from 2% to 88% and averaging from 10% to 22% resulting in an additional cost of $70,000 per infant (Jadcherla et al., 2013; Slaughter et al., 2016). GERD is commonly managed using proton pump inhibitors (PPI) to reduce gastric acid secretion (El‐Mahdy et al., 2017; Yadlapati & Kahrilas, 2018). Symptoms often persist leading to parental/provider refusal to discontinue use and prolonged treatment. Long‐term use of PPI can result in unintended consequences of aerodigestive infections, nutrient malabsorption, and bone fractures (Gulati & Jadcherla, 2019). Additional research on esophageal pathophysiology is necessary to refine precise diagnostic and management strategies based on pathophysiologic mechanisms in high‐risk infants.

With increased esophageal acid exposure, mucosal permeability increases (Borrelli et al., 2012; Cohen Sabban et al., 2017; Jadcherla et al., 2019). Distal baseline impedance (DBI), measured via 24‐h pH‐impedance testing, has been used as a surrogate marker of mucosal permeability (Jadcherla et al., 2019; Zhong et al., 2013). As ascertained from older children, DBI <900 Ω has been associated with severe esophagitis (biopsy score of 3), and children with DBI >2000 Ω had biopsy scores of 0 to 2 or no endoscopic evidence of esophagitis (Cohen Sabban et al., 2017). It is believed that inflammation plays a role in the progression of gastroesophageal reflux (GER) to GERD, but the pathophysiology and natural course as well as the mechanistic basis for symptoms remain unclear in high‐risk infants. Although we do not know how esophageal inflammation develops or its direct effect on esophageal function, there is data from human and animal models in that, with prolonged exposure to acidic material, the normal processes of the epithelium, muscular layers, and nerves are affected (Lang et al., 2019; Page et al., 2002; Ribolsi et al., 2020; Szczesniak et al., 2008). Damage to the esophageal layers may be responsible for nociceptive mechanisms, symptoms, and signs (Mittal, 2016).

We have previously characterized provocation induced mid‐esophageal reflexes during maturation and disease states in infants to investigate physiologic and pathophysiologic pharyngo‐esophageal motility mechanisms (Hill & Jadcherla, 2013; Jadcherla et al., 2005; Jadcherla et al., 2006; Jadcherla, Shubert, Malkar, et al., 2015; Pena et al., 2010). Additionally, we have evaluated and compared pH‐impedance characteristics in high‐risk infants classified by DBI thresholds (<900 Ω, 900–2000 Ω, and >2000 Ω), wherein it was reported that those infants with DBI <900 Ω had prolonged acid exposure, delayed clearance (based on bolus clearance times), and greater aerodigestive symptoms (Jadcherla et al., 2019). We concluded that clarification of dysmotility mechanisms may provide guidance for personalized therapies (Jadcherla et al., 2019). Therefore, our aim was to compare sensory‐motor characteristics of mid‐esophageal provocation induced motility reflexes and clinical outcomes among DBI categories (<900 Ω vs. 900‐2000 Ω vs. >2000 Ω). We hypothesized that mid‐esophageal provocation induced reflexes are modified in infants with impaired esophageal permeability.

## 2 MATERIALS AND METHODS

### 2.1 Participants, setting, study design

The reported findings are from secondary analysis of our prior clinical study in infants with GERD (Jadcherla, Hasenstab, Gulati, et al., 2020). Infants were included if they met the criteria: (a) Acid reflux exposure >3% confirmed by 24‐h pH‐impedance testing, (b) evaluated between 34 and 60 weeks postmenstrual age, and (c) had absence of genetic, metabolic, or syndromic diseases, severe neuropathology, gastroesophageal malformations, or surgical conditions. For the current study goals, they also underwent pharyngo‐esophageal motility testing to characterize the sensory‐motor properties of the reflexes. Studies were performed at the Innovative Infant Feeding Disorders Research Program at Nationwide Children's Hospital. Prior to enrollment, Institutional Review Board approval (IRB11‐00734) and informed parental consent were obtained. Health insurance portability and accountability act guidelines were followed.

### 2.2 Testing protocols

#### 2.2.1 pH‐impedance

Infants underwent 24‐h pH‐impedance testing via a disposable pH‐impedance probe with six impedance channels and one distal pH sensor (Greenfield MMS‐Z1‐I or ZandorpH MMS‐Z1‐P‐7R, Laborie Medical Technologies) connected to a recording device (MMS Ohmega, Laborie Medical Technologies) (Jadcherla et al., 2019; Jadcherla, Sultana, et al., 2020). The probe was calibrated with buffer solutions of pH 4.0 and pH 7.0 before placement. After testing, data was downloaded and analyzed using analysis software (MMS Version 9.6). Subjects were classified by average baseline impedance value (ohms) at the most distal impedance channel into the following groups: DBI <900 Ω, DBI between 900 and 2000 Ω, or DBI >2000 Ω (Borrelli et al., 2012; Cohen Sabban et al., 2017; Jadcherla et al., 2019).

#### 2.2.2 Pharyngo‐Esophageal motility

Following the pH‐impedance test, infants underwent water perfusion pharyngo‐esophageal motility testing as previously validated. Briefly, a 6 Fr silicone catheter (Dentsleeve International, Mui Scientific) with a mid‐esophageal infusion port for infusing stimuli, ports for manometric recording of pharyngeal, esophageal (proximal, middle, and distal), and gastric responses, as well as sleeves for upper esophageal sphincter (UES) and lower esophageal sphincter (LES) responses was connected to a water perfusion motility system (Solar GI; Laborie Medical Technologies) (Jadcherla, Hasenstab, Gulati, et al., 2020; Jadcherla, Shubert, Gulati, et al., 2015). The catheter was positioned by the study physician using the pull through technique, so that upper and lower esophageal sphincters were at the position of the sleeves. Infant's respiratory changes were monitored continuously and concurrently using respiratory inductance plethysmography (Respitrace, Viasys) and nasal airflow thermistor (Integra Life Sciences) (Jadcherla et al., 2007; Jadcherla, Hasenstab, et al., 2015). Additionally, patient safety and symptoms were monitored by the study physician and registered nurse at the patient's bedside. Mid‐esophageal stimulation included graded doses of 0.1, 0.5, 1, and 2 ml of media air, water, and apple juice (pH of 3.5) to simulate gas, non‐acid liquid, and acid liquid GER, respectively, in triplicate.

### 2.3 Data analysis

#### 2.3.1 Esophageal motility characteristics

Sensory‐motor characteristics of responses to mid‐esophageal stimulation were evaluated for peristaltic response, UES function, esophageal body function, LES function, respiratory rhythm changes, and symptoms as follows: (a) *Response occurrence* (%) as the presence of esophago‐deglutition reflex (EDR) characterized by pharyngeal contraction, UES relaxation, esophageal peristalsis, and LES relaxation (Gupta et al., 2009; Jadcherla & Rudolph, 2005), secondary peristalsis (SP) characterized by UES contraction, esophageal peristalsis, and LES relaxation (Gupta et al., 2009; Jadcherla et al., 2006), UES contractile reflex (UESCR) defined as an increase ≥4 mmHg from baseline (Jadcherla, Hasenstab, et al., 2015; Jadcherla et al., 2006), esophageal body polymorphic waveforms characterized by multiple esophageal peaks (Hill & Jadcherla, 2013), LES relaxation (LESR) defined as a decrease ≥5 mmHg from baseline (Jadcherla, Hasenstab, et al., 2015; Pena et al., 2010), respiratory rhythm change (Jadcherla, 2012; Jadcherla, Hasenstab, et al., 2015), and symptoms, (b) *response latency*, s, as the time duration from the start of the infusion to the start of the response, and (c) *response magnitude* defined as the pressure, mmHg, and duration, s, for UESCR, esophageal body (proximal, middle, and distal segments), LESR, and respiratory rhythm changes (Jadcherla, 2012; Jadcherla, Hasenstab, et al., 2015), and (d) threshold volume, ml, defined as the volume needed to induce either a primary response, UESCR, or LESR (Jadcherla et al., 2006).

#### 2.3.2 Statistical analysis

Data were compared among the three DBI categories: <900 Ω, 900–2000 Ω, and > 2000 Ω. Chi‐square or fisher exact tests were used, as appropriate, to analyze categorical variables. Kruskal–Wallis test or analysis of variance (ANOVA) were used to analyzed continuous variables. Generalized Estimating Equations (GEEs) with repeated measurements were applied to examine the relationships between the distal baseline impedance category and different clinical outcomes. All models were adjusted to media (air, water, apple juice) and volume (0.1, 0.5, 1, and 2 ml) effects and their interaction (if significant). To account for dependency in repeated measures the compound symmetric correlation was used. Logit and cumulative logit link were used for categorical outcomes, identity and logarithm link were used for continuous outcomes. Bonferroni adjustment was applied for multiple comparisons between the three DBI categories. Linear regression and a Pearson correlation coefficient were implemented to assess the relationship between DBI and ARI and longest reflux event by the presence of chronic lung disease (CLD). SAS (version 9.4) was used to perform the analysis. A *p*‐value of ≤0.05 was considered statistically significant.

## 3 RESULTS

### 3.1 Clinical characteristics of participants & aerodigestive outcomes

**FIGURE 1:**
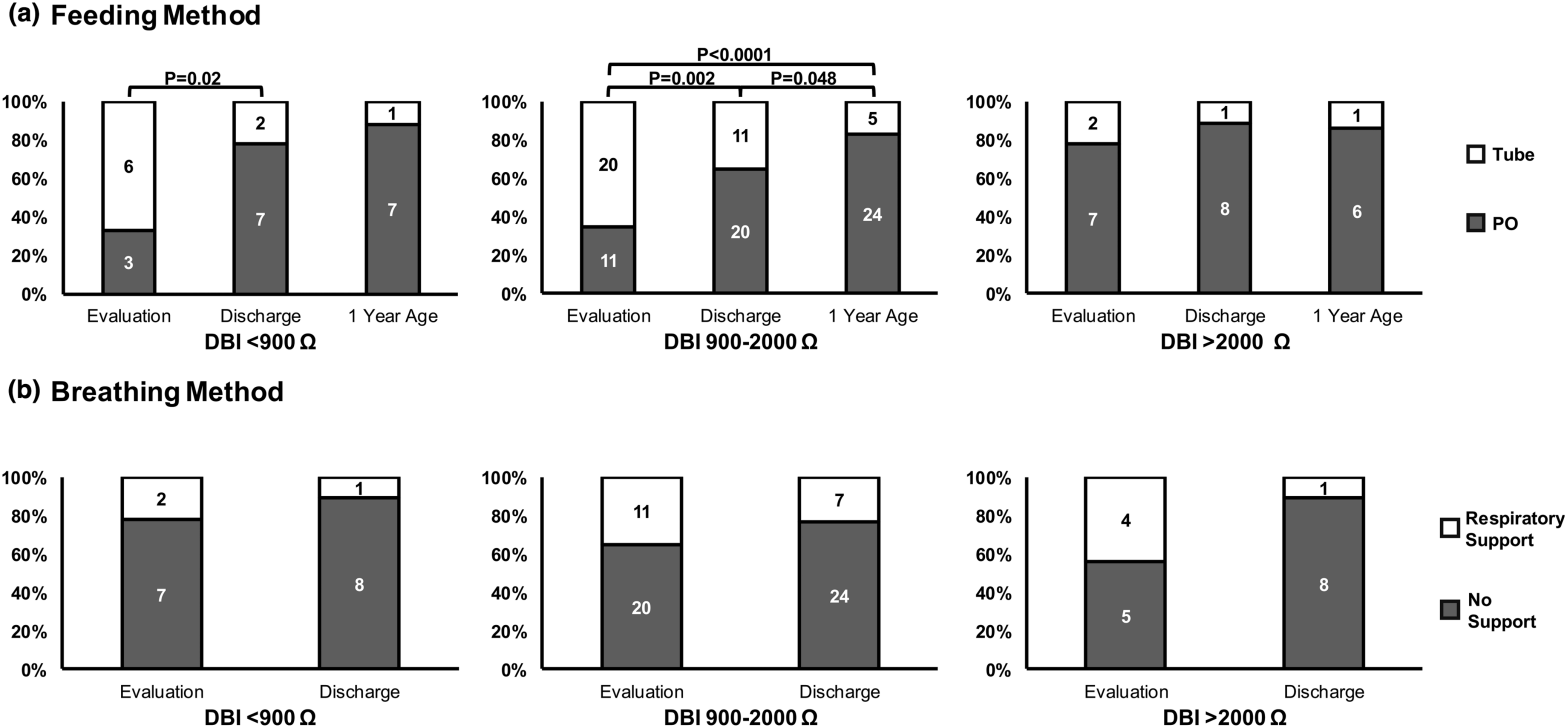
Oral feeding and breathing outcomes among DBI groups. Comparisons were performed within and between DBI groups, with patient n‐values denoted by numbers inside the bars. There were no significant differences between DBI groups (all *p* > 0.05). (a) Feeding methods‐ Tube defined as any tube feeding; PO defined as independent oral feeding. Despite the severity of mucosal permeability, majority of infants were achieving full oral feeding at either discharge or 1‐year of age (*p* < 0.05). Also, note that most of the infants in the <900 Ω and 900–2000 Ω groups improved between evaluation and discharge. (b) Breathing methods‐ Respiratory support defined as nasal cannula oxygen. No support defined as room air. Although there were no distinct differences with maturation or between DBI groups, note that >77% of infants in any DBI group were not on any respiratory support at discharge. Data from DBI <900 Ω (*n* = 9), DBI 900–2000 Ω (*n* = 31), and DBI >2000 Ω (*n* = 9) are shown for evaluation and discharge. However, at 1‐year age, the data includes DBI <900 Ω (*n* = 8), DBI 900–2000 Ω (*n* = 29), and DBI >2000 Ω (*n* = 7).

Comparison of participant (*N* = 49) characteristics at birth, at evaluation, at discharge and at 1‐year age that underwent mid‐esophageal provocation testing among DBI categories are shown (Table 1). Importantly, note that most characteristics did not significantly differ between groups. However, infants with DBI <900 Ω had increased acid exposure (ARI) and minimal prevalence of CLD, while infants with DBI >2000 Ω had decreased acid exposure and an increased prevalence of CLD (*p* < 0.05). Feeding and breathing methods at evaluation and discharge classified by DBI categories are shown in Figure 1. Although there were no differences between DBI categories for feeding and breathing methods, infants with DBI <900 Ω and 900–2000 Ω had increased need for feeding tube support at evaluation (*p* < 0.05), which improved with time.

**TABLE 1:** Comparison of clinical and demographic outcomes by distal baseline impedance

Characteristic	Distal baseline impedance category			Overall *p*‐value
	<900 Ω (*N* = 9 infants)	900–2000 Ω (*N* = 31 infants)	>2000 Ω (*N* = 9 infants)	
At birth				
Gender, male, *n* (%)	3 (33%)	16 (52%)	6 (67%)	0.37
Ethnicity, non‐Hispanic, *n* (%)	9 (100%)	29 (94%)	9 (100%)	0.99
Race, *n* (%)				
African American or Black	1 (11%)	6 (19%)	3 (33%)	0.86
White	7 (78%)	20 (65%)	6 (67%)	
Other	1 (11%)	4 (13%)	0 (0%)	
Unknown	0 (0%)	1 (3%)	0 (0%)	
Gestational age, weeks	31.4 ± 4.6	31.1 ± 4.3	27.6 ± 3.5	0.09
Birth weight, kg	1.75 ± 1.15	1.81 ± 1.20	1.11 ± 5.94	0.15
Birth length, cm	41.1 ± 7.7	40.9 ± 7.1	36.0 ± 5.4	0.17
Birth head circumference, cm	27.4 ± 5.2	28.1 ± 4.1	25.0 ± 3.5	0.21
APGAR at 1 min, score	6 ± 2, *n* = 8	5 ± 3, *n* = 29	4 ± 2, *n* = 8	0.18
APGAR at 5 min, score	8 ± 2	7 ± 2	7 ± 2	0.25
Morbidities				
Preterm birth, *n* (%)	7 (78%)	26 (84%)	9 (100%)	0.45
Neuropathology, *n* (%)	3 (33%)	10 (32%)	5 (56%)	0.43
Chronic lung disease, *n* (%)	2 (22%)	15 (48%)	8 (89%)*	**0.02**
At evaluation				
Postmenstrual age, weeks	41.1 ± 2.8	41.4 ± 2.7	41.1 ± 2.4	0.91
Weight, kg	3.16 ± 7.99	3.65 ± 9.04	3.59 ± 6.87	0.32
Length, cm	48.4 ± 4.5	49.6 ± 4.1	49.2 ± 2.4	0.71
Head circumference, cm	33.9 ± 1.7	34.5 ± 2.1	34.9 ± 2.0	0.60
ARI, %	22.3 ± 16.4	9.9 ± 5.4	8.8 ± 5.3 *	**0.03**
ARI (Indeterminate: Abnormal), %	(11%: 89%)	(35%: 65%)	(44%: 56%)	0.35
At discharge				
Weight, kg	4.14 ± 6.78	4.56 ± 1.07	4.31 ± 7.48	0.47
Length, cm	53.2 ± 2.9	54.0 ± 3.8	52.0 ± 3.8	0.32
Head circumference, cm	36.5 ± 1.4	36.5 ± 2.2	36.6 ± 1.5	0.99
Length of hospital stay, days	68 (31, 152)	67 (8, 222)	23 (6, 166)	0.58
At 1‐year age				
Emergency room visits, *n* (%)	4 (44%)	13 (42%)	4 (44%)	0.99
Readmissions, *n* (%)	1 (11%)	11 (35%)	4 (44%)	0.34

*Note*: Data presented as n (%), mean ± SD, or median (IQR).

Abbreviations: ARI, Acid Reflux Index.

*p < 0.05 vs. DBI <900 Ω.

### 3.2 Effect of DBI category on characteristics of esophageal reflexes

**FIGURE 2:**
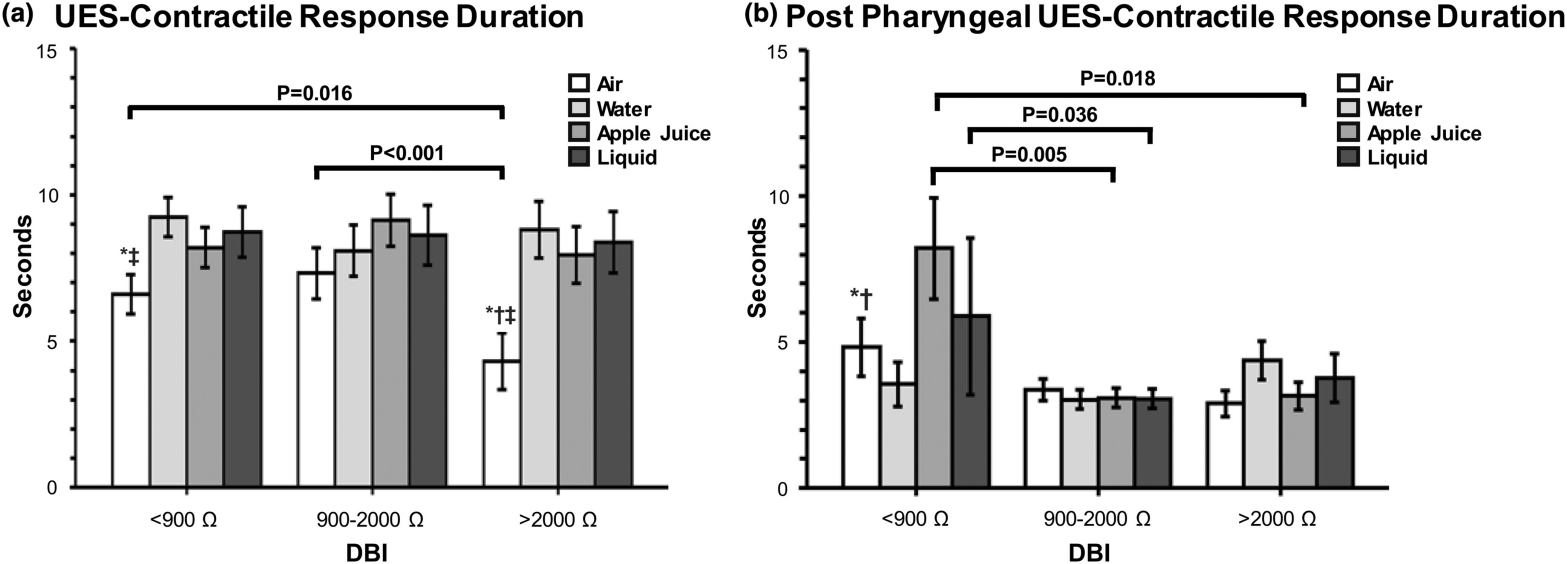
DBI and stimulus media interactions for UES contractile response characteristics. *p < 0.05 versus water, †*p* < 0.05 versus apple juice, ‡*p* < 0.05 versus liquid. Note that (a) UES‐contractile response duration occurring with secondary peristalsis is prolonged with air in 900–2000 Ω and <900 Ω DBI groups, indicating increased mechano‐sensitivity, whereas (b) UES‐contractile response occurring after esophago‐deglutition reflex is more in the <900 Ω DBI group with apple juice.

**FIGURE 3:**
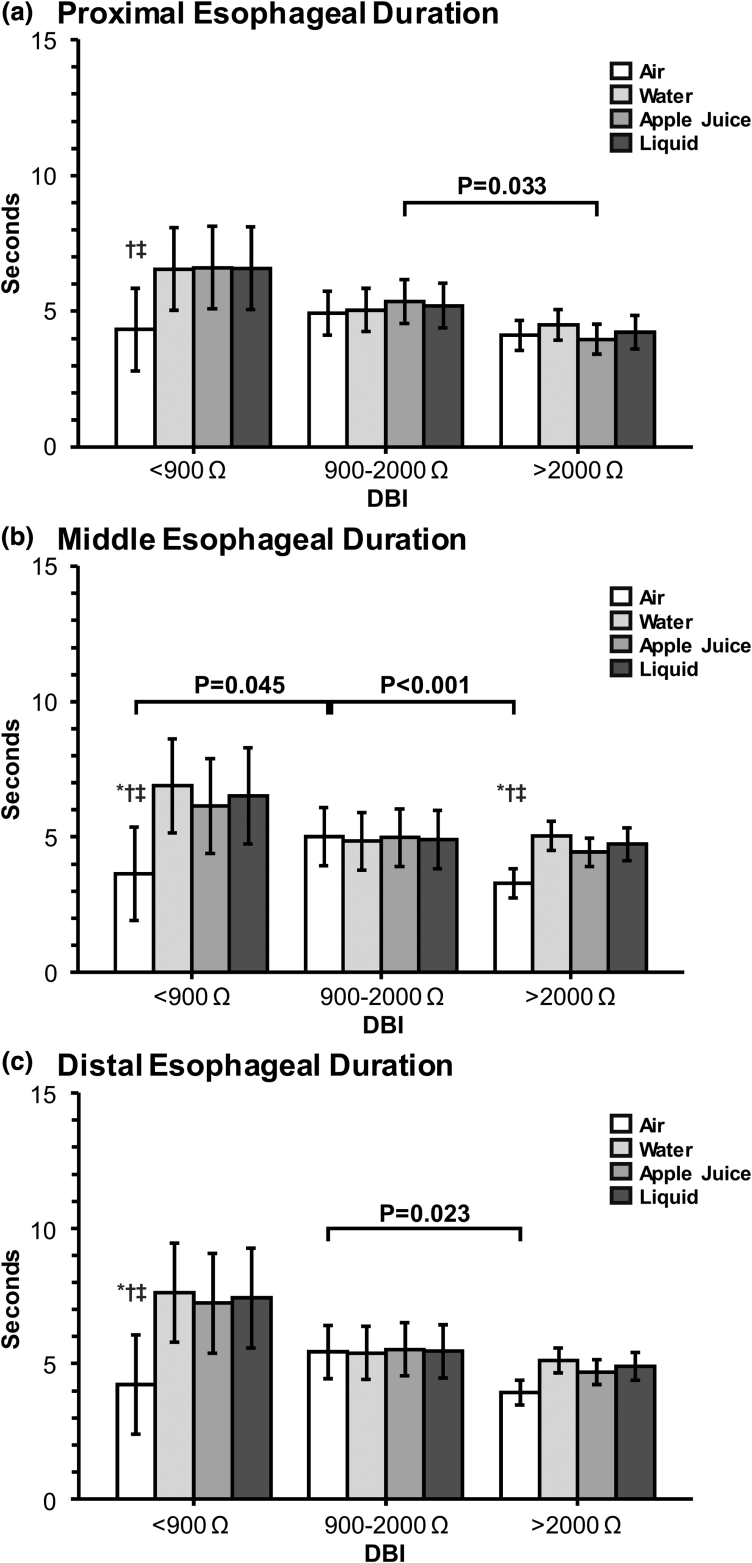
DBI and stimulus media interactions for esophageal contractile characteristics. *p < 0.05 versus water, †*p* < 0.05 versus apple juice, ‡*p* < 0.05 versus liquid. With DBI <900 Ω, esophageal contraction durations at proximal, middle, and distal esophageal segments between air and liquid were distinct (*p* < 0.05), suggesting differences in mechano‐ and chemo‐sensitive effects. Note that in the proximal esophagus, the differences were noted with apple juice whereas the middle and distal esophagus had differences in air between the DBI groups.

Baseline esophageal sphincter characteristics (UES or LES) did not differ except for infants in the DBI 900–2000 Ω group who had on average a 5.7 mmHg increase in resting pressure versus the DBI >2000 Ω group (*p* = 0.02). Comparison of response occurrence for overall peristaltic, UES, esophageal, LES, respiratory responses, and symptoms between DBI groups are shown in Table 2. Note, the increased prevalence of polymorphic waveforms in infants with DBI <900 Ω and DBI 900–2000 Ω, indicating esophageal dysmotility. Comparison of pharyngo‐esophageal sensitivity characteristics between DBI groups are shown in Table 3. Notable was decreased peristaltic sensory threshold volumes (indicating greater sensitivity) for air in infants with DBI 900–2000 Ω, and for sterile water in infants with DBI <900 Ω. Comparison of esophageal response magnitudes between DBI groups are shown in Table 4. No significant differences were noted. Interactions between stimulus media and DBI for UES contractile reflexes (Figure 2), as well as proximal‐, middle‐, and distal‐ esophageal contractile characteristics (Figure 3) are shown. Note the mechano‐ and chemo‐ sensitive effects in infants with impaired mucosal permeability. Lastly, we performed correlation analysis of respiratory and digestive motility variables while keeping the DBI as a continuous variable (Appendix 1); no strong correlations were noted.

**TABLE 2:** Comparison of mid‐esophageal infusion induced response occurrence by distal baseline impedance

Characteristic		Distal baseline impedance category (OR 95% CI)			Overall *p*‐value
		900–2000 Ω vs. <900 Ω	<900 Ω vs. >2000 Ω	900–2000 Ω vs. >2000 Ω	
Overall peristaltic response					
**Peristaltic response**	*Adjusted*	1.1 (0.8, 1.7)	1.4 (0.7, 2.9)	1.6 (0.8, 3.3)	0.23
	*Unadjusted*	1.2 (0.8, 1.7)	1.4 (0.7, 2.6)	1.6 (0.8, 3.0)	0.21
**Response type, EDR vs. SP**	*Adjusted*	1.6 (0.6, 4.6)	0.8 (0.2, 3.1)	1.3 (0.5, 3.5)	0.53
	*Unadjusted*	1.5 (0.5, 3.9)	0.8 (0.2, 3.1)	1.2 (0.4, 3.3)	0.63
**Multiple peristaltic response**	*Adjusted*	1.0 (0.7, 1.6)	1.1 (0.6, 2.0)	1.1 (0.6, 2.1)	0.92
	*Unadjusted*	1.0 (0.6, 1.6)	1.1 (0.6, 2.0)	1.1 (0.6, 2.0)	0.96
UES					
**Contractile reflex with SP**	*Adjusted*	0.6 (0.3, 1.6)	1.7 (0.6, 4.6)	1.1 (0.5, 2.2)	0.44
	*Unadjusted*	0.7 (0.4, 1.4)	1.5 (0.7, 3.0)	1.1 (0.7, 1.8)	0.41
**Contractile reflex after EDR**	*Adjusted*	1.6 (0.5, 5.1)	0.6 (0.2, 2.4)	0.9 (0.2, 4.1)	0.52
	*Unadjusted*	1.3 (0.4, 4.1)	0.7 (0.2, 3.0)	0.9 (0.2, 4.0)	0.79
Esophagus					
**Complete peristalsis**	*Adjusted*	0.6 (0.2, 1.6)	0.5 (0.2, 1.8)	0.3 (0.1, 0.9)*	**0.02**
	*Unadjusted*	0.7 (0.3, 1.7)	0.5 (0.1, 1.5)	0.3 (0.1, 0.8)*	**0.01**
**Polymorphic waveform**	*Adjusted*	0.8 (0.4, 1.7)	2.5 (1.1, 5.9)*	2.0 (1.1, 3.8)*	**0.01**
	*Unadjusted*	0.8 (0.4, 1.7)	2.3 (1.0, 5.3)*	1.8 (1.0, 3.3)*	**0.02**
LES					
**Relaxation reflex**	*Adjusted*	0.8 (0.4, 1.5)	1.7 (0.7, 4.3)	1.3 (0.6, 2.8)	0.32
	*Unadjusted*	0.7 (0.4, 1.2)	1.6 (0.8, 3.1)	1.1 (0.6, 2.1)	0.17
**SP**	*Adjusted*	0.5 (0.2, 1.5)	1.2 (0.3, 4.0)	0.5 (0.2, 1.4)	0.15
	*Unadjusted*	0.5 (0.2, 1.4)	1.0 (0.4, 3.1)	0.5 (0.2, 1.3)	0.13
**EDR**	*Adjusted*	0.3 (0.1, 1.6)	2.8 (0.5, 16.2)	0.9 (0.3, 3.0)	0.23
	*Unadjusted*	0.3 (0.1, 1.4)	2.8 (0.5, 14.3)	0.8 (0.3, 2.5)	0.17
Respiratory					
**Rhythm change**	*Adjusted*	1.1 (0.7, 1.8)	1.2 (0.6, 2.2)	1.2 (0.7, 2.3)	0.69
	*Unadjusted*	1.1 (0.7, 1.7)	1.2 (0.6, 2.2)	1.2 (0.7, 2.2)	0.67
Symptoms					
**Symptom**	*Adjusted*	0.8 (0.5, 1.2)	1.1 (0.6, 1.9)	0.8 (0.5, 1.3)	0.34
	*Unadjusted*	0.8 (0.5, 1.3)	1.1 (0.6, 1.9)	0.8 (0.5, 1.3)	0.42

*Note*: Data is presented as adjusted and unadjusted Odds Ratio (95% CI). The adjusted model includes stimulus volume and stimulus media effects.

Abbreviations: EDR, esophago‐deglutition response, SP, secondary peristalsis.

* *p*‐value <0.05 considered significant. The overall *p*‐value refers to the hypothesis that there is no difference between the three DBI groups for each variable. If the overall *p*‐value is not significant it means that no differences were found among the three groups. If the overall *p*‐value is significant, then post‐hoc comparisons (Bonferroni adjustment) were made to assess inter‐group significance represented by the bold text and the asterisk. The underlined distal baseline impedance category represents the reference group. As an example, for polymorphic waveforms there was a significant difference found among the three DBI groups. Furthermore, comparison showed that polymorphic waveforms are more likely to occur in <900 Ω and 900–2000 Ω groups versus the >2000 Ω group.

**TABLE 3:** Comparison of sensory metrics during esophageal infusion induced responses by distal baseline impedance

Characteristic	Distal baseline impedance category			Overall *p*‐value
	<900 Ω (*N* = 9 infants)	900–2000 Ω (*N* = 31 infants)	>2000 Ω (*N* = 9 infants)	
Peristaltic response				
Overall TV, ml	0.4 ± 0.1	0.4 ± 0.1, *n* = 30	0.7 ± 0.2	0.26
Air TV, ml	0.4 ± 0.1, *n* = 7	0.3 ± 0.1*, *n* = 28	0.8 ± 0.2	**0.02**
Liquid TV, ml	0.8 ± 0.1	1.0 ± 0.1	1.5 ± 0.2	0.11
Water TV, ml	0.2 ± 0.1*†, *n* = 8	0.5 ± 0.1, *n* = 29	0.8 ± 0.2	**0.04**
Apple Juice TV, ml	0.6 ± 0.1	0.6 ± 0.1, *n* = 29	0.8 ± 0.2, *n* = 8	0.67
Response latency, s	3.8 ± 0.3	3.8 ± 0.2	4.1 ± 0.3	0.69
UES				
Contractile reflex TV, ml	0.9 ± 0.2	1.0 ± 0.1	1.1 ± 0.2	0.72
Air TV, ml	1.4 ± 0.6, *n* = 7	0.8 ± 0.1, *n* = 25	0.9 ± 0.2, *n* = 6	0.62
Liquid TV, ml	1.1 ± 0.2	1.9 ± 0.2	2.0 ± 0.3	0.08
Water TV, ml	0.8 ± 0.2, *n* = 8	0.9 ± 0.1, *n* = 26	1.3 ± 0.2, *n* = 7	0.31
Apple Juice TV, ml	0.6 ± 0.1, *n* = 6	1.2 ± 0.1, *n* = 28	1.1 ± 0.2, *n* = 8	0.10
Contractile reflex latency, s	4.3 ± 0.4	3.7 ± 0.2	4.1 ± 0.3	0.20
Esophagus				
Response latency, s	8.1 ± 1.1	7.3 ± 0.3	6.4 ± 0.5	0.23
LES				
Relaxation reflex TV, ml	0.4 ± 0.1	0.5 ± 0.1, *n* = 30	0.5 ± 0.1	0.65
Relaxation reflex latency, s	5.3 ± 0.5	5.1 ± 0.2	5.4 ± 0.3	0.78
Respiratory				
Response latency, s	5.2 ± 0.5	5.4 ± 0.2	5.1 ± 0.4	0.75

*Note*: Data presented as LSM ± SE.

Infants were excluded if they did not receive a specific media infusion or if the average media threshold volume could not be calculated, which can happen in the absence of a response.

Abbreviations: TV, response threshold volume.

* *p* < 0.05 vs. DBI >2000 Ω.

† *p* < 0.05 vs. DBI 900–2000 Ω.

**TABLE 4:** Comparison of mid‐esophageal infusion induced response magnitudes by distal baseline impedance

Characteristic		Distal baseline impedance category effect (95% CI)			Overall *p*‐value
		900–2000 Ω vs. <900 Ω	<900 Ω vs. >2000 Ω	900–2000 Ω vs. >2000 Ω	
Overall response					
^2^Total peristaltic responses	*Adjusted*	1.0 (0.9, 1.2)	1.0 (0.9, 1.2)	1.0 (0.9, 1.3)	0.85
	*Unadjusted*	1.0 (0.9, 1.2)	1.0 (0.9, 1.2)	1.0 (0.9, 1.2)	0.88
^2^Total response time	*Adjusted*	0.9 (0.7, 1.2)	1.0 (0.7, 1.3)	0.9 (0.7, 1.1)	0.44
	*Unadjusted*	0.9 (0.7, 1.2)	1.0 (0.7, 1.4)	0.9 (0.7, 1.1)	0.46
UES					
^1^Contractile pressure with SP	*Adjusted*	5.4 (−10.7, 21.6)	0.5 (−19.7, 20.6)	5.9 (−8.6, 20.4)	0.50
	*Unadjusted*	5.7 (−10.1, 21.6)	−0.6 (−20.3, 19.1)	5.2 (−9.3, 19.6)	0.53
^2^Contractile magnitude with SP	*Adjusted*	0.7 (0.3, 1.7)	0.7 (0.3, 1.9)	0.5 (0.2, 1.5)	0.34
	*Unadjusted*	0.6 (0.3, 1.4)	0.9 (0.4, 2.1)	0.6 (0.3, 1.3)	0.19
^1^Contractile pressure after EDR	*Adjusted*	−7.8 (−33.9, 18.3)	19.7 (−10.7, 50.0)	11.9 (−7.5, 31.3)	0.22
	*Unadjusted*	−5.4 (−30.5, 19.7)	15.0 (−15.5, 45.4)	9.6 (−11.3, 30.4)	0.44
Esophagus					
^1^Proximal esophagus amplitude	*Adjusted*	2.1 (−8.5, 12.6)	−3.3 (−19.1, 12.6)	−1.2 (−15.5, 13.1)	0.86
	*Unadjusted*	1.9 (−8.5, 12.4)	−3.3 (−19.0, 12.5)	−1.3 (−15.7, 13.0)	0.86
^1^Middle esophagus amplitude	*Adjusted*	−2.9 (−11.5, 5.8)	6.6 (−4.4, 17.6)	3.8 (−9.2, 16.7)	0.30
	*Unadjusted*	−2.9 (−11.2, 5.5)	6.4 (−4.5, 17.3)	3.5 (−9.3, 16.3)	0.30
^1^Distal esophagus amplitude	*Adjusted*	8.7 (−0.7, 18.1)	−2.4 (−12.1, 7.3)	6.3 (−5.5, 18.1)	0.08
	*Unadjusted*	8.5 (−0.8, 17.8)	−2.4 (−12.3, 7.5)	6.1 (−5.8, 17.9)	0.09
LES					
^1^Relaxation pressure	*Adjusted*	−0.6 (−3.8, 2.6)	2.1 (−1.8, 5.9)	1.5 (−1.7, 4.6)	0.40
	*Unadjusted*	−0.7 (−3.8, 2.4)	2.3 (−1.6, 6.1)	1.6 (−1.6, 4.7)	0.34
^1^Relaxation magnitude	*Adjusted*	8.6 (−6.1, 23.4)	−19.4 (−44.0, 5.1)	−10.8 (−35.3, 13.7)	0.11
	*Unadjusted*	8.8 (−5.8, 23.4)	−19.9 (−44.3, 4.4)	−11.2 (−35.5, 13.2)	0.10
^2^Relaxation onset to nadir	*Adjusted*	1.0 (0.7, 1.3)	0.8 (0.5, 1.3)	0.8 (0.6, 1.2)	0.38
	*Unadjusted*	1.0 (0.7, 1.4)	0.8 (0.5, 1.3)	0.8 (0.6, 1.2)	0.42
^2^Relaxation duration	*Adjusted*	1.1 (0.8, 1.6)	0.9 (0.6, 1.2)	1.0 (0.8, 1.3)	0.62
	*Unadjusted*	1.1 (0.8, 1.6)	0.9 (0.6, 1.2)	1.0 (0.8, 1.3)	0.57
Respiratory					
^2^Response duration	*Adjusted*	0.8 (0.6, 1.1)	1.1 (0.7, 1.6)	0.8 (0.6, 1.2)	0.14
	*Unadjusted*	0.8 (0.5, 1.1)	1.1 (0.7, 1.6)	0.8 (0.5, 1.2)	0.10

*Note*: Data presented as unadjusted and adjusted Effect (95% CI).

Abbreviations: EDR, esophago‐deglutition response; SP, secondary peristalsis.

^1^ The estimate represents LS‐mean difference.

^2^ The estimate represents the relative ratio (exponentiated coefficient)—based on logarithm link. The adjusted model includes stimulus volume and stimulus media effects. Interactions are included if found significant. *p‐value <0.05 considered significant. The overall *p*‐value refers to the hypothesis that there is no difference between the three DBI groups for each variable. If the overall *p*‐value is not significant it means that no differences were found among the three groups. If the overall *p*‐value is significant, then post‐hoc comparisons (Bonferroni adjustment) were made to assess inter‐group significance represented by the bold text and the asterisk. The underlined distal baseline impedance category represents the reference group. There are no significant differences in response magnitudes between the DBI groups.

### 3.3 Effect of chronic lung disease on DBI relationships

**FIGURE 4:**
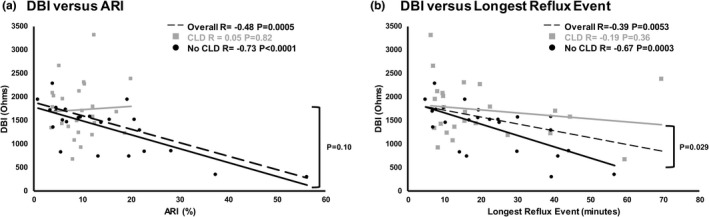
Effects of chronic lung disease (CLD) in infancy on distal baseline impedance (DBI) relationships with esophageal acid exposure. Comparisons were made between CLD and no CLD groups, DBI versus acid reflux index (ARI), and DBI versus longest reflux event. (a) DBI versus ARI relationships: In infants without CLD (*n* = 24), DBI decreases with ARI increase, whereas there was no correlation of DBI and ARI in infants with CLD (*n* = 25), or a difference between the two groups, indicating that ARI is the driving variable in the DBI value, not CLD. (b) DBI versus longest reflux duration relationships: In the infants without CLD, DBI decreases with longer events, whereas there was no correlation for the CLD group.

The correlation between DBI and ARI severities and the longest reflux event in those with and without CLD are shown (Figure 4). Note the correlation of DBI with ARI and longest reflux event in infants without CLD, but not in infants with CLD.

## 4 DISCUSSION

### 4.1 Summary

The salient observations of our study are: (1) In infants where DBI was <900 Ω or between 900 and 2000 Ω (vs. >2000 Ω), we noted: (a) A greater prevalence of tube feeding, although the majority were able to achieve independent oral feeding by discharge or 1 year (Figure 1a). (b) Esophageal reflexes were impaired as noted by the decreased prevalence of complete peristalsis and increased prevalence of polymorphic waveforms (Table 2). (c) Sensitivity thresholds were lower for peristaltic responses with mid‐esophageal air and water stimulation (Table 3). (d) Mechano‐stimulation with air resulted in prolonged UESCR associated with SP responses (Figure 2a), and with liquid or acidic (apple juice) stimulation, the duration for post pharyngeal UESCR associated with EDR responses was increased (Figure 2b). (2) In infants where DBI was <900 Ω, contractions within the entire esophagus were prolonged with liquid stimuli versus air (Figure 3). (3) ARI and DBI were correlated in infants without CLD, as consistent with previous studies (Borrelli et al., 2012; Cohen Sabban et al., 2017; Jadcherla et al., 2019; Ribolsi et al., 2020), but were not correlated in infants with CLD.

To our knowledge, this is the first study in human infants to examine the effect of mucosal permeability thresholds (DBI <900 Ω, 900–2000 Ω, and >2000 Ω) on sensory‐motor characteristics of pharyngo‐esophageal and sphincteric reflexes. We tested the hypothesis that impairments in esophageal mucosal permeability (DBI <900 Ω and 900–2000 Ω) altered the response to mid‐esophageal stimulus, and that the evoked reflex characteristics worsen based on the severity of DBI thresholds (such as in <900 Ω category). We concluded that the frequency‐occurrence and sensory‐motor characteristics of pharyngo‐esophageal motility reflexes in human infants are modifiable based on esophageal permeability thresholds.

### 4.2 Physiological and pathophysiological considerations

#### 4.2.1 Esophageal sensitivity

Abnormalities of pharyngo‐esophageal motility and clearance may play a role in the GERD diagnosis and/or generation of symptoms. Although not proven in infants, but confirmed in adults with GERD, acid, and non‐acid GER events have been shown to alter peristaltic and upper esophageal sphincter contractile reflexes (UESCR) (Savarino et al., 2011). In prior studies with adult animal and adult human models, the effects of acid exposure on UES reflexes were examined (Lang et al., 2019; Szczesniak et al., 2008). In the animal model, UES relaxation was more sensitive (Lang et al., 2019), while in the human model UES contraction was more frequent (Szczesniak et al., 2008). Additionally, in animals, esophageal acid exposure increases sensitization in mucosal mechanoreceptors (Page et al., 2002). However, these studies did not examine the effects of esophageal mucosal permeability levels or its impact on LES function. In our study, esophageal peristalsis and UES functions were modified, while LES function was maintained in infants with DBI <900 Ω. Specifically, mechano‐ and osmo‐receptors are more sensitive in generating an overall peristaltic response. Depending on the magnitude of esophageal acid exposure or upon increased mucosal permeability, infants may be less sensitive to acid and more sensitive to gas and non‐acid liquid GER. Additionally, UES contractile reflexes indicate hypervigilant or hypersensitive states providing sustained protection against ascending refluxate. Increased durations of UES and esophageal body contractions lead to longer esophageal clearance time indicating dysmotility at the proximal‐, middle‐, and distal‐ esophageal levels.

The UES and proximal esophageal (skeletal muscle) contractile characteristics were different at low DBI states; persistence of such effects may be due to sensitization of afferent and efferent nerves in the proximal aerodigestive region. Thus, a trivial stimulus (low threshold) can magnify reflexes that are prolonged (heightened sensitivity and extended stimulation) along with symptom occurrence. Multiple symptom‐generating mechanisms may exist, such as those mediated by mechanosensitive‐, chemo‐ or osmo‐ sensitive receptors, any of which may contribute to the development of visceral hyperalgesia in premature infants which manifests as persistent symptoms and signs that are indeed reflexes at the skeletal and smooth muscle level. Potential therapeutic targets may therefore depend on the underlying mechanisms.

#### 4.2.2 Peristaltic functions

In a study of adults responding to PPI therapy, lower DBI was associated with absent esophageal peristalsis, but not fragmented peristalsis (Ribolsi et al., 2020). In contrast, in our study, infants with low DBI had incomplete or failed peristalsis, as well as polymorphic waveforms (Table 2). These findings may be due to prolonged and weak excitatory effects or inadequate inhibitory effects at the esophagus; it has been shown that inflammation in the gut modifies both excitatory and inhibitory signaling activities at the smooth muscle and skeletal muscle levels (Collins, 1996; Jacobson et al., 1995; Jadcherla, 2002; Lomax et al., 2005; Verne et al., 2001). However, despite DBI <900 Ω, most infants were able to achieve successful oral feeding, indicating that dysmotility mechanisms may be reversible in neonates.

### 4.3 Clinical and translational significance

Infants recuperating in the NICU frequently suffer from aerodigestive comorbidities related to systemic or regional inflammatory states such as prematurity, hypoxia, chronic lung disease, and/or GERD. Within the neonatal population, heterogeneity exists, and the role of coexisting factors in modifying pharyngo‐esophageal sensory motor characteristics need a larger study. Our study population included preterm and full‐born infants, with or without mild neuropathology, with or without mild to moderate chronic lung disease (<2 liters per minute of oxygen), and were studied prior to any surgical interventions. Those with CLD did not follow correlation patterns between ARI and DBI as noted before (Borrelli et al., 2012; van der Pol et al., 2013; Zhong et al., 2013). A recent retrospective study in infants with bronchopulmonary dysplasia had significantly lower DBI values compared to those without lung disease; however, the DBI values were still >2000 Ω (Nobile et al., 2022), which is consistent with our findings. Whether this observation is related to coexistent airway‐digestive‐nutritive therapies needs further investigation.

Airway or digestive inflammatory states can alter sensitivity and motor responses, which may manifest as aerodigestive symptoms. In infants, DBI had been shown to improve with PPI therapy, but symptoms did not improve (Loots et al., 2012). Recently, we have shown that in infants treated with PPI, perception of symptom burden decreased (Jadcherla, Hasenstab, Wei, et al., 2020), but pharyngeal‐esophageal motility mechanisms worsened (Jadcherla, Hasenstab, Gulati, et al., 2020). It is unknown if PPI improves esophageal motility and symptoms in infants with DBI <900 Ω. On the other hand, underlying esophageal motility disturbances could be primary (maturational delays), leading to altered DBI due to retained and prolonged bolus presence distally. Nevertheless, based on our preliminary findings, more focused physiological testing in carefully selected, homogeneous populations are needed to clarify the observed associations between mucosal permeability changes and esophageal dysmotility.

### 4.4 Limitations

Our clinical study has limitations but provides support for further investigations: (1) Infants with DBI <900 Ω and DBI ≥2000 Ω were of small sample size. Therefore, the data should be considered preliminary, hypothesis‐generating, and needs further prospective study. This study may form the basis for more focused physiological testing including clinical or pathological evaluation (esophageal tissue examination) where permitted. Furthermore, investigation in animal models may inform approaches towards delineating causal and curative mechanisms during inflammation and or maturation. (2) DBI was used as a biomarker of mucosal permeability as esophageal biopsies are not feasible in infants. Additionally, acute mucosal permeability changes could not be detected without the use of a third catheter specifically designed for the detection of mucosal conductivity (as it is ethically not permissible). (3) Currently, there are no standard DBI thresholds for high‐risk infants. Therefore, we utilized the DBI thresholds based on the threshold values of older children (median age 7.4) (Cohen Sabban et al., 2017). (4) It is possible that poor esophageal clearance and fluid stasis due to esophageal dysmotility or ineffective esophageal motility could lead to decreased DBI values (as in infants who are tube fed). Thus, further studies are needed to distinguish causal effects.

## 5 CONCLUSIONS

High‐risk infants with DBI <900 Ω may have heightened visceral sensitivity, esophageal dysfunction (ineffective esophageal peristalsis characterized by absent or incomplete propagation or polymorphic waveforms) and exaggerated upper aerodigestive protection (UES contractile reflexes). Additionally, infants with DBI between 900 and 2000 Ω may be more sensitive to gas stimulus, and infants with DBI of <900 Ω may be more sensitive to non‐acid liquid stimulus. These increased mucosal permeability categories are associated with alterations of sensory‐motor enteric and central nervous system reflexes that are triggered even with trivial stimulus and may represent early onset of visceral hyperalgesia states. Our study findings lead the way for further physiological testing in convalescing high‐risk infants to ascertain potential mechanisms of airway‐digestive reflex interactions and symptom generation, which may lead to precise therapies.

## AUTHOR CONTRIBUTIONS

Sudarshan R. Jadcherla sought IRB approval and obtained grant funding. Sudarshan R. Jadcherla, Roseanna Helmick, and Kathryn A. Hasenstab were involved with the study design and performed studies. Sudarshan R. Jadcherla, Roseanna Helmick, and Minna Njeh developed the first manuscript draft. Roseanna Helmick and Kathryn A. Hasenstab performed motility data analysis. Enas Alshaikh performed statistical analysis. Roseanna Helmick, Kathryn A. Hasenstab, Minna Njeh, and Enas Alshaikh prepared figures and tables. Sudarshan R. Jadcherla, Roseanna Helmick, Kathryn A. Hasenstab, Minna Njeh, and Enas Alshaikh verified data, edited, and revised manuscript, and approved final version as submitted.

## FUNDING SOURCE

Supported in part by: a) the National Institutes of Health grants by the National Institute of Diabetes and Digestive and Kidney Diseases (R01 DK 068158 [to S.R.J.]), and National Center for Advancing Translational Sciences (UL1TR002733 [to The Ohio State University Center for Clinical and Translational Science for REDCap support]), and b) the International Foundation for Gastrointestinal Disorders Research Excellence Award [to S.R.J.].

## DISCLOSURE STATEMENT

The authors have no conflicts of interest to declare.

## Appendix 1 Correlation of DBI and pharyngo‐esophageal motility characteristics

**Table:**

Characteristic	*r*‐value	*p*‐value
Peristaltic response		
Response latency	0.01	0.77
Total peristaltic responses	−0.02	0.55
Total response time	−0.05	0.09
UES		
Contractile reflex latency	−0.03	0.38
Contractile pressure with SP	0.12	<0.01
Contractile magnitude with SP	0.05	0.17
Contractile pressure after EDR	−0.04	0.51
Esophagus		
Response latency	−0.11	<0.01
Response duration	−0.05	0.06
Proximal esophagus amplitude	0.04	0.25
Middle esophagus amplitude	−0.06	0.05
Distal esophagus amplitude	−0.01	0.82
LES		
Response latency	0.02	0.60
Relaxation pressure	−0.06	0.03
Relaxation magnitude	0.11	<0.01
Relaxation onset to nadir	0.04	0.20
Relaxation duration	0.02	0.47
*Respiratory*		
Response latency	−0.07	0.07
Response duration	−0.04	0.25
